# Targeting Coagulation Factor Xa Promotes Regression of Advanced Atherosclerosis in Apolipoprotein-E Deficient Mice

**DOI:** 10.1038/s41598-019-40602-w

**Published:** 2019-03-07

**Authors:** Jelle J. Posthuma, Jens J. N. Posma, Rene van Oerle, Peter Leenders, Rick H. van Gorp, Armand M. G. Jaminon, Nigel Mackman, Stefan Heitmeier, Leon J. Schurgers, Hugo ten Cate, Henri M. H. Spronk

**Affiliations:** 1 Laboratory for Clinical Thrombosis and Haemostasis, Department of Internal Medicine, Cardiovascular Research Institute Maastricht, Maastricht University Medical Centre, Maastricht, The Netherlands; 2 Department of Surgery, Amsterdam University Medical Center, Location AMC, Amsterdam, The Netherlands; 3 Department of Pharmacology-Toxicology, Cardiovascular Research Institute Maastricht, Maastricht University, Maastricht, The Netherlands; 4 Department of Biochemistry, Cardiovascular Research Institute Maastricht, Maastricht University, Maastricht, The Netherlands; 5 Thrombosis and Hemostasis Program, Division of Hematology and Oncology, Department of Medicine, University of North Carolina at Chapel Hill, Chapel Hill, North Carolina USA; 6 Research & Development,Pharmaceuticals, Bayer AG, Wuppertal, Germany

## Abstract

Atherosclerosis is a progressive inflammatory vascular disorder, complicated by plaque rupture and subsequently atherothrombosis. *In vitro* studies indicate that key clotting proteases, such as factor Xa (FXa), can promote atherosclerosis, presumably mediated through protease activated receptors (PARs). Although experimental studies showed reduced onset of atherosclerosis upon FXa inhibition, the effect on pre-existing plaques has never been studied. Therefore, we investigated effects of FXa inhibition by rivaroxaban on both newly-formed and pre-existing atherosclerotic plaques in apolipoprotein-e deficient (ApoE^−/−^) mice. Female ApoE^−/−^ mice (age: 8–9 weeks, n = 10/group) received western type diet (WTD) or WTD supplemented with rivaroxaban (1.2 mg/g) for 14 weeks. In a second arm, mice received a WTD for 14 weeks, followed by continuation with either WTD or WTD supplemented with rivaroxaban (1.2 mg/g) for 6 weeks (total 20 weeks). Atherosclerotic burden in aortic arch was assessed by haematoxilin & eosin immunohistochemistry (IHC); plaque vulnerability was examined by IHC against macrophages, collagen, vascular smooth muscle cells (VSMC) and matrix metalloproteinases (MMPs). In addition, PAR1 and -2 expressions and their main activators thrombin and FXa in the plaque were determined in the plaque. Administration of rivaroxaban at human therapeutic concentrations reduced the onset of atherosclerosis (−46%, *p* < *0.05)*, and promoted a regression of pre-existing plaques in the carotids (−24%, *p* < *0.001*). In addition, the vulnerability of pre-existing plaques was reduced by FXa inhibition as reflected by reduced macrophages (−39.03%, *p* < *0.05*), enhanced collagen deposition (+38.47%, *p* < *0.05*) and diminished necrotic core (−31.39%, *p* < *0.05*). These findings were accompanied with elevated vascular smooth muscle cells and reduced MMPs. Furthermore, expression of PARs and their activators, thrombin and FXa was diminished after rivaroxaban treatment. Pharmacological inhibition of FXa promotes regression of advanced atherosclerotic plaques and enhances plaque stability. These data suggest that inhibition of FXa may be beneficial in prevention and regression of atherosclerosis, possibly mediated through reduced activation of PARs.

## Introduction

Atherosclerosis is a multifactorial disease, characterized by progressive chronic inflammation of the arterial wall^1^, starting in response to lipid accumulation and subsequent inflammation in the arterial wall and together drive the formation of an atherosclerotic plaque[1]. Disruption of atherosclerotic plaque is the underlying cause of luminal thrombosis (atherothrombosis), responsible for most acute coronary syndromes^2^. Several studies showed that the plaque phenotype, a thin fibrous cap, large necrotic core, and presence of macrophages, determine atherosclerotic plaque vulnerability for rupturing and thus the risk of atherothrombosis^2–5^. The exact processes contributing to plaque instability are poorly understood.

Accumulating evidence shows that key coagulation enzymes, such as factor Xa (FXa) and thrombin, can influence a wide range of cellular actions related to cardiovascular function, such as vascular permeability, inflammation, and apoptosis. These non-haemostatic actions are predominantly mediated through activation of protease-activated receptors (PARs)^6–9^. PARs belong to the family of G protein-coupled receptors. To date, four PARs have been identified, PAR1 to -4, which are expressed on a variety of cell types involved in atherosclerosis, including endothelial cells (EC), vascular smooth muscle cells (VSMC), fibroblasts, T lymphocytes, and monocytes^9–11^. PAR1, -3 and -4 are predominantly activated by thrombin, whereas FXa activates PAR1, -2 and -3 (alone or in complex with tissue factor, factor VIIa)^7,12,13^. Experimental animal studies demonstrated that transgenic mice carrying the TM^pro/pro^ and ApoE^−/−^ genes, resulting in respectively a hypercoagulable and pro-atherogenic phenotype, developed more vulnerable atherosclerotic plaques^14^. In contrast, administration of direct thrombin inhibitors in atherogenic mice attenuates atherosclerotic plaque formation and promotes plaque stability by reducing inflammation, which is accompanied by a reduced expression of PAR1^14,15^. Similarly inhibition of FXa, utilizing a low dose of rivaroxaban, had a beneficial effect in ApoE^−/−^ mice in terms of plaque stability and inflammation^16^. In contrast, Hara *et al*. (2015) reduced the plaque formation, stabilized the plaque and decreased inflammation with a low dose of rivaroxaban in ApoE^−/−^ mice. Additionally they showed decreased MMP-9 expression^17^.

Experimental studies using either a FXa or thrombin inhibitor focused on newly-formed atherosclerosis instead of the clinically relevant treatment of already developed atherosclerosis. We therefore studied the effects of FXa inhibition on pre-excising atherosclerotic plaques utilizing optimal direct FXa inhibitor plasma levels.

We hypothesize that pharmacologic inhibition of FXa at human therapeutic levels of rivaroxaban reduces progression of pre-existing atherosclerotic plaques in a mouse model for atherosclerosis.

## Materials and Methods

### Animals

Female C57BL-6 ApoE^−/−^ mice (Charles River, Maastricht, The Netherlands) were used throughout all experiments. Animals were housed in a temperature-controlled environment with a 12 h light/12 h dark cycle. All animal experimental protocols were approved by the Institutional Animal Care and Use Committee of Maastricht University (Maastricht, The Netherlands) and all protocols were carried out in compliance with the Dutch government guidelines and the guidelines from Directive 2010/63/EU of the European Parliament on the protection of animals used for scientific purposes.

### Plaque progression models and pharmacological interventions

**Figure 1:**
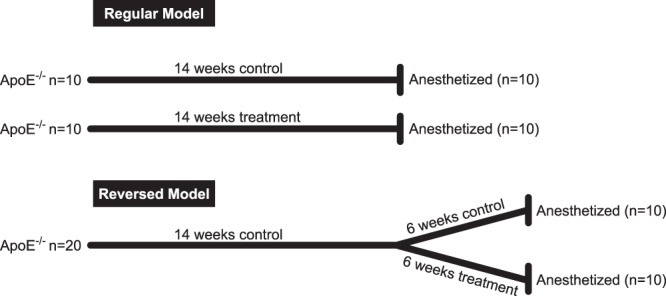
Animal model of regression. In the regular model, animals were either put on regular WTD as a control or WTD supplemented with rivaroxaban for 14 weeks. In our reversed model, all animals received WTD during the first 14 weeks. After 14 weeks, the group was divided in 2:1 group continued with WTD for the remainder of 6 weeks, and one group was switched to WTD supplemented with rivaroxaban.

Female ApoE^−/−^ mice (age, 8–9 weeks) were fed a western type diet (WTD) *ab libitum* throughout the experiments (15% cocoa butter, 1% corn oil, 0.25% cholesterol, 40.5% sucrose, 10% cornstarch, 20% casein, free of cholate, total fat content 16%; ABdiets, Woerden, The Netherlands). In a pilot study, therapeutic rivaroxaban levels (150–350 ng/mL) were reached with WTD supplemented with 1.2 mg/g rivaroxaban. In the first arm, female ApoE^−/−^ mice (n = 10/group) received WTD or WTD supplemented with rivaroxaban (1.2 mg/g) for 14 weeks. In our second arm, termed regression model, mice (n = 20) received initially a WTD for 14 weeks without rivaroxaban treatment. After 14 weeks, these mice were randomly divided in 2 equal groups (n = 10/group): 1 group received WTD for 6 weeks and 1 group received WTD supplemented with rivaroxaban (1.2 mg/g) for 6 weeks to investigate the effects of FXa inhibition on pre-existing atherosclerotic. After experiments, all mice were anaesthetized with inhaled isoflurane (2.3%) and sacrificed using pentobarbital overdose for detailed analysis (Fig. 1). In addition to this, blood was collected without fasting directly after sacrificing the mice, for further blood analysis.

### Thrombin generation

Thrombin generation in plasma was measured by means of the Calibrated Automated Thrombography (CAT) method (Thrombinoscope BV, Maastricht, The Netherlands), employing a low affinity fluorogenic thrombin substrate (Z-Gly-Gly-Arg-amino-metyl-coumarin) to continuously monitor thrombin activity in clotting plasma. Measurements were conducted in 10 µL of 3.2% (w/v) citrated plasma in a total volume of 120 µL as described previously[16]. Coagulation was triggered by adding 4 µM phospholipid vesicles (phosphatidyl serine/phosphatidyl ethanolamine/phosphatidyl choline, 20:20:60) and 1 pM tissue factor, followed by 14.5 mM (final concentrations) CaCl_2_. In order to correct for inner-filter effects and substrate consumption, each thrombin generation measurement was calibrated against the fluorescence curve obtained in a sample from the same plasma, added with a fixed amount of thrombin-α2-macroglobulin complex (Thrombin Calibrator, Thrombinoscope BV, Maastricht, The Netherlands). Fluorescence was read in a Fluoroskan Ascent reader (Thermo Labsystems OY, Helsinki, Finland) equipped with a 390/460 filter set and thrombin generation curves were calculated with Thrombinoscope software (Thrombinoscope BV, Maastricht, The Netherlands). The curves were automatically analyzed for lag time, thrombin peak height, and endogenous thrombin potential (ETP; area under the thrombin generation curve).

### Determination of lipid levels and rivaroxaban concentration

Plasma concentrations of total cholesterol, triglycerides (TGL), high-density lipoprotein (HDL) and low-density lipoprotein (LDL) were determined enzymatically in 3.2% (w/v) citrated plasma with a Cobas 8000 analyzer (Roche Diagnostics, Almere, The Netherlands). Rivaroxaban concentrations were measured in plasma based on a FXa dependent substrate hydrolysis reaction utilizing a Biophen DiXal kit (Aniara, Hyphen biomed) on an automatic coagulation analyser (BCS-xp, Siemens Diagnostics Products Corporation, Marburg, Germany).

### Histological and morphometric analysis

Aortic arches and carotid arteries of mice were acquired at the end of the experiment, fixed in formalin (10%) embedded in paraffin. Paraffinized aortic arches were cut in tissue sections of 5 µm. For immunohistochemical staining, tissue sections were dewaxed, rehydrated, and subsequently stained with hematoxylin and eosin (HE) (Klinipath, Duiven, The Netherlands) for morphometric analysis. Quantification of the atherosclerotic content in the aortic arch was performed by staining longitudinal sections of the aortic arch at 20 µm interval with H&E. Sections in which maximal lesion size was observed were used to measure the total surface of atherosclerotic plaques within the lumen side of the aortic arch.

For antibody-based immunohistochemical staining, endogenous peroxidase activity in aortic arches was inhibited with hydrogen peroxide (0.33% in methanol; Merck Millipore, Billerica, USA), and tissues were incubated with antigen retrieval solution (Sigma-Aldrich, St. Louis, USA) for 30 minutes and blocked with 5% normal serum in TBST pH = 7.4 for 60 minutes. Sections were then incubated overnight with primary antibodies in Tris-Buffered Saline 0.1% Tween (TBST, pH = 7.4) and 3% normal serum. Applied primary antibodies: anti-factor X antibody (Novus Biologicals, Littleton, USA), anti-thrombin antibody (Novus Biologicals, Littleton, USA), anti-factor VII antibody (Novus Biologicals, Littleton, USA), anti-PAR1 antibody (Bioss Inc., Woburn, USA), anti-PAR2 antibody (Abcam, Cambridge, UK), anti-tissue factor antibody (Abcam, Cambridge, UK), anti-collagen type 1 antibody (Novus Biologicals, Littleton, USA), anti-alpha smooth muscle actin antibody (Abcam, Cambridge, UK), anti-MAC2 antibody (Abcam, Cambridge, UK). Sections were incubated with biotinylated secondary antibodies for 45 minutes followed by 60 minutes incubation in ABC complex (vectastain elite ABC HRP kit, Vector Laboratories, USA) according to manufacturers’ protocol. For visualization ImmPACT NovaRED (Vector Bio-connect) was used. Collagen was visualized by Picrosirius red staining (Sigma-Aldrich, St. Louis, USA), calcification by alizarin red S staining (Sigma-Aldrich, St. Louis, USA) and necrotic core by toluidin blue staining (Sigma-Aldrich, St. Louis, USA), all according to the manufacturers’ protocols. The extent of positive staining within the lesions was determined with ImageJ Software (National Institutes of Health, USA) in duplicate by operators blinded for treatment allocation.

### Statistical analysis

Statistical analysis was performed using Prism version 7 (GraphPad Software Inc., San Diego, CA, USA) and IBM SPSS statistics 23.0 (SPSS Japan Inc., an IBM company, Tokyo, Japan). All data were analyzed using a Mann-Whitney U test. Data are shown as difference compared to 14 weeks or as median (IQR), unless otherwise stated. A 2-tailed p < 0.05 was considered as statistically significant.

## Results

### Inhibition of FXa reduces thrombin generation and does not affect body weight or plasma lipid profile in ApoE^−/−^ mice

All mice treated with rivaroxaban reached therapeutic plasma levels (median: 210 ng/mL, range: 150–260 ng/mL). Treatment with rivaroxaban reduced *ex vivo* thrombin generation as reflected by increased lag time compared to controls (14 weeks: +56%, *p* < *0.0001*, and 20 weeks: +60%, *p* < *0.001*) and reduced ETP (14 weeks: −11%, *p* < *0.05*, and 20 weeks: −9%, *p* < *0.05*), whereas no statistically significant differences were observed in peak height (Table 1). During the entire experimental period, no bleeding complications were observed in mice. In addition, administration of rivaroxaban had no significant effects on body weight, total cholesterol, TGL, LDL, and HDL (Table 1).

**Table 1:** Effects of rivaroxaban on body weight, metabolic profile and thrombin generation.

	14 weeks WTD	14 weeks WTD + Riva	*p-value*	20 weeks WTD	*p-value*	20 weeks WTD + Riva	*p-value*
Body weight (g)	22 (21–24)	23 (22–25)	*0.3726*	24 (23–26)	0.0446*	24 (23–25)	0.0138*
Rivaroxaban level (ug/L)		217 (156–349)				240 (150–247)	
**Lipid profile**							
Total cholesterol (mmol/L)	18.25 (16.72–23.58)	20.71 (19.50–23.40)	*0.4000*	24.62 (22.10–27.67)	*0.1111*	22.50 (20.20–25.13)	*0.3429*
TGL (mmol/L)	0.47 (0.39–0.63)	0.42 (0.41–0.47)	*0.5714*	0.62 (0.48–0.74)	*0.4603*	0.58 (0.50–0.62)	*0.4857*
LDL (mmol/L)	15.35 (13.10–22.70)	16.80 (16.50–23.20)	*0.3456*	19.90 (18.00–23.10)	*0.4524*	17.30 (16.65–22.23)	*0.3429*
HDL (mmol/L)	5.15 (3.22–5.52)	6.45 (4.73–7.45)	*0.4238*	6.15 (2.67–6.72)	*0.2857*	5.89 (4.95–7.89)	*0.4429*
**Thrombin generation**							
Lag time (min)	2.08 (1.82–2.29)	3.24 (2.61–3.50)	<0.0001*	2.33 (2.00–2.37)	*0.1322*	3.33 (3.08–3.46)	0.0002*
Peak height (nmol/L)	71.65 (67.02–82.01)	67.88 (64.21–69.59)	*0.0684*	89.40 (82.54–94.51)	0.0127*	71.71 (61.70–77.72)	*0.7023*
ETP (nmol/L.min)	451 (413–476)	498 (405–574)	0.0322*	456 (411–534)	*0.8272*	409 (376–431)	0.0432*

Note: All values are median(IQR), *p < 0.05 vs 14 weeks WTD, n = 10/group.

Abbreviations: TGL = Triglyceride, HDL = high-density lipoprotein, LDL = low-density lipoprotein, ETP = endogenous thrombin potential.

### Coagulation is a key factor in the onset of atherosclerosis

**Figure 2:**
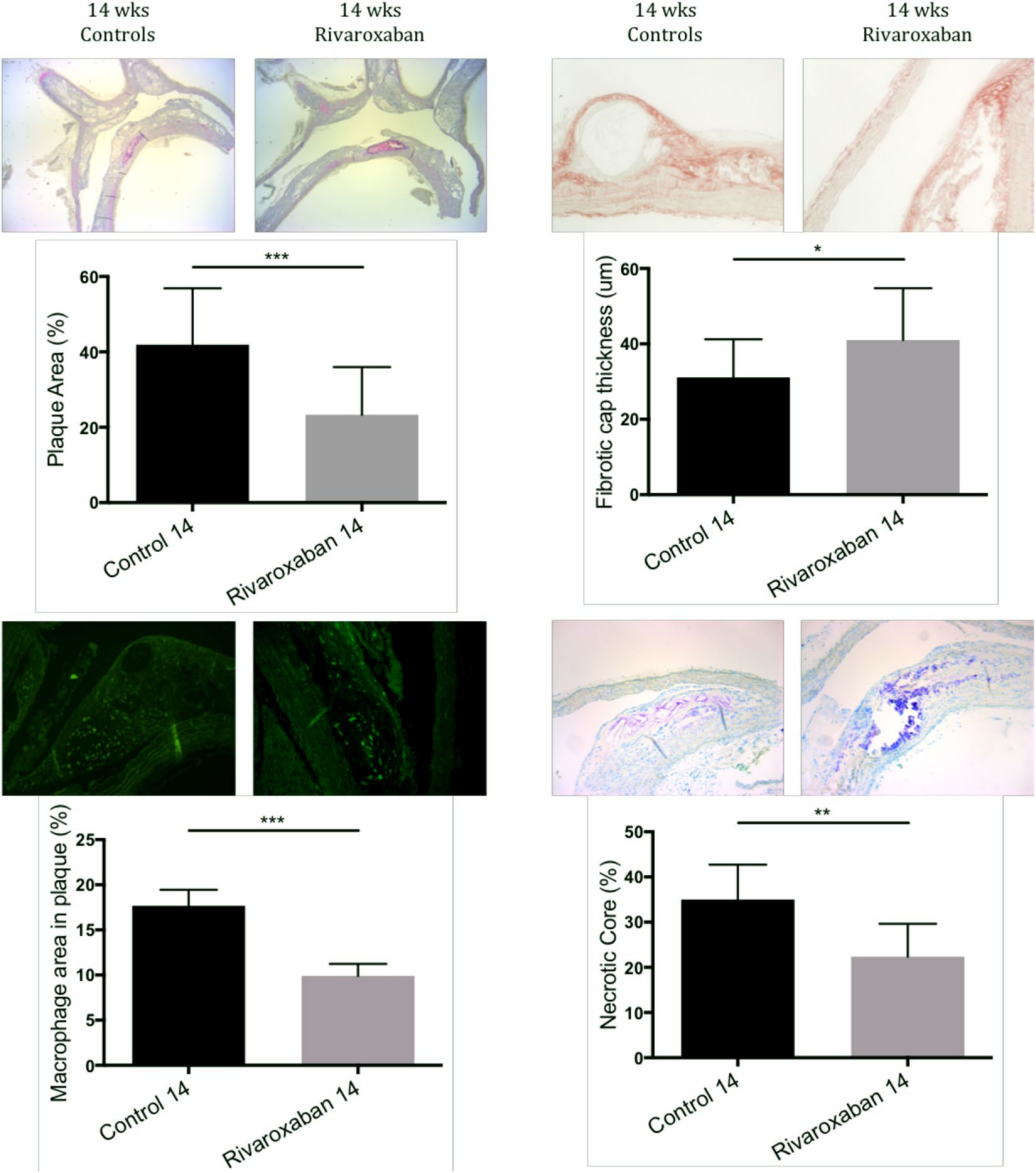
Plaque development in the aortic arch of ApoE^−/−^ mice. The aortic arch of rivaroxaban treated mice showed less plaque formation than control (−46%). This was associated with a more stable phenotype of the plaque as measured by a thicker fibrotic cap (29.50 μm (26.00–40.50)) compared to control (41.00 μm (33.25–54.75)), reduced macrophages (17.68% (13.25–19.46) vs 9.90% (9.23–11.24), and decreased necrotic core (34.56% (27.85–40.63) vs 22.92% (14.94–28.43). *p < 0.05, **p < 0.01, ***p < 0.001. All data were in median (IQR) n = 10 for each group.

Quantitative analysis of atherosclerotic lesions in the aortic arch revealed a significant reduced onset of atherosclerosis in mice receiving rivaroxaban for 14 weeks, as compared to WTD (−46%, *p* = *0.001*) (Fig. 2). The findings were accompanied by a more stable plaque phenotype, as reflected by increased fibrotic cap thickness (29.50 μm (26.00–40.50) vs 41.00 μm (33.25–54.75), *p* < *0.05*), reduced macrophages staining (17.68% (13.25–19.46) vs 9.90% (9.23–11.24), *p* < *0.001*) and a smaller necrotic core (34.56% (27.85–40.63) vs 22.92% (14.94–28.43), *p* < *0.01*).

### Inhibition of FXa induces regression of advanced atherosclerotic lesions

**Figure 3:**
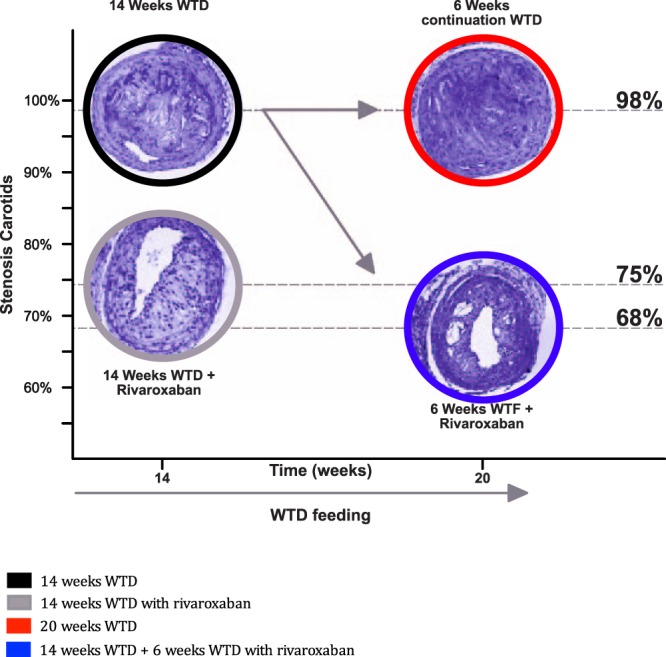
Plaque regression in the carotids of ApoE^−/−^ mice. (**A**) Carotids stained with hematoxilin & eosin (H&E), used to quantify the extent of atherosclerotic plaque in the luminal side after 14 and 20 weeks. (**B**) Quantitative analysis of area of atherosclerotic plaque in aortic arch lumen after 14 weeks WTD: 96.48% (87.27–99.33) vs WTD + rivaroxaban: 74.88% (60.83–80.60) and 20 weeks: 98.01% (96.45–99.56) vs 67.66% (56.43–80.97). All data were in median (IQR), n = 10 for each group.

In the second arm, after an initial period of 14 weeks WTD, 6 weeks of rivaroxaban treatment at human therapeutic levels significantly reduced pre-existing atherosclerotic plaques (−24%, *p* < *0.001*), whereas plaque size remained equal in the control group compared to 14 weeks (+10%, *p* = *0.41*) (Fig. 3).

### Inhibition of FXa increases atherosclerotic plaque stability in advanced lesions

**Figure 4:**
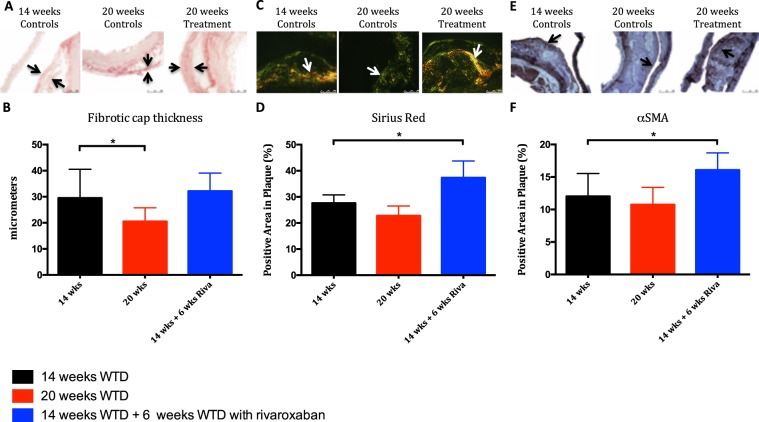
Enhanced plaque stability in ApoE^−/−^ regression mice treated with rivaroxaban. (**A**)Top row represents images of atherosclerotic plaque in the aortic arch stained with sirius red, used to quantify total collagen of atherosclerotic plaque under polarized light after 14 and 20 weeks. (**B**) Quantitative analysis of total collagen in atherosclerotic plaque after 14 weeks: 27.62% (23.79–30.76) and 20 weeks: 22.65% (20.73–25.65) vs 37.03% (31.24–43.21). (**C**)Atherosclerotic plaque in the aortic arch, stained with sirius red, used to quantify the mean fibrotic cap thickness of atherosclerotic plaque after 14 and 20 weeks. (**D**) Quantitative analysis of mean fibrotic cap thickness after 14 weeks: 29.50 μm (26.00–40.50) and 20 weeks: 20.00 μm (15.50–25.75) vs 35.00 μm (28.00–38.00). (**E**) Top row represents images of atherosclerotic plaque in the aortic arch stained against αSMA, used to quantify the expression of VSMC in atherosclerotic plaques. (**F**) Quantitative analysis of αSMA expression in atherosclerotic plaques after 14 weeks: 12.02% (10.83–15.55) and 20 weeks: 9.98% (8.98–11.91) vs 16.11% (13.95–19.04). All data were median (IQR), *p < 0.05 vs 14 weeks, n = 10 for each group.

The regressed atherosclerotic plaques in the second arm of the study were associated with enhanced plaque stability in rivaroxaban treated mice. This was reflected by elevated total collagen (+35%, *p* < *0.01*) in plaques of mice treated with rivaroxaban from 14 to 20 weeks, where total collagen tended to decrease in mice receiving WTD for 20 weeks (−18%, *p* = *0.06*). Furthermore, mean fibrotic cap thickness reduced from 14 to 20 weeks in control mice (−33%, p < 0.05), while rivaroxaban preserved the fibrotic cap thickness after 6 weeks of treatment (+19%, *p* = *0.82*). These findings were supported by increased VSMCs levels (αSMA positive area, +33%, *p* < *0.05*) after treatment with rivaroxaban, compared to WTD only (−17%, *p* = *0.11)* (Fig. 4) and reduced collagen degradation proteins MMP9 and −13 (−45%, *p* = *0.02* and −36%, *p* = *0.01*).

### Targeting FXa reduces inflammation and necrotic core in pre-existing plaques

**Figure 5:**
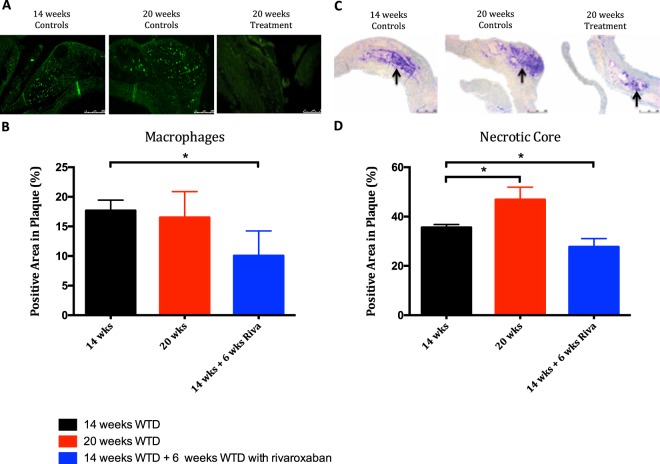
Reduced inflammation and necrotic core in rivaroxaban treated ApoE^−/−^ regression mice. (**A**) Atherosclerotic plaques in the aortic arch, stained against macrophages by Mac2, used to quantify the infiltration of macrophages in atherosclerotic plaque after 14 and 20 weeks. (**B**) Quantitative analysis of Mac2 after 14 weeks: 17.68% (13.25–19.46) and 20 weeks: 16.54% (12.39–20.42) vs 9.96% (6.20–13.44). (**C**) Top row represents images of atherosclerotic plaque in the aortic arch stained with toluidin blue, used to quantify the size of necrotic core within atherosclerotic plaques after 14 and 20 weeks. (**D**) Quantitative analysis of toluidin blue in atherosclerotic plaque after 14 weeks: 35.58% (33.73–36.75) and 20 weeks: 48.00% (42.00–51.00) vs. 26.84% (25.00–31.00) All data were median (IQR), *p < 0.05 vs 14 weeks, n = 10 for each group.

Treatment with rivaroxaban decreased inflammation in pre-existing atherosclerotic plaques, as revealed by reduced expression of macrophages (mac2 positive area) (−44%, *p* = *0.009*), while inflammation remained unchanged in mice receiving WTD from 14 to 20 week (−6%, *p* > *0.9999*). These findings were accompanied by a significantly reduced necrotic core upon rivaroxaban treatment (−25%, *p* = *0.001*), which increased upon continuation with WTD for 6 weeks (+35%, *p* = *0.0028)* (Fig. 5).

### Reduced expression of coagulation factors and PAR 1 and 2 in atherosclerotic lesions

**Figure 6:**
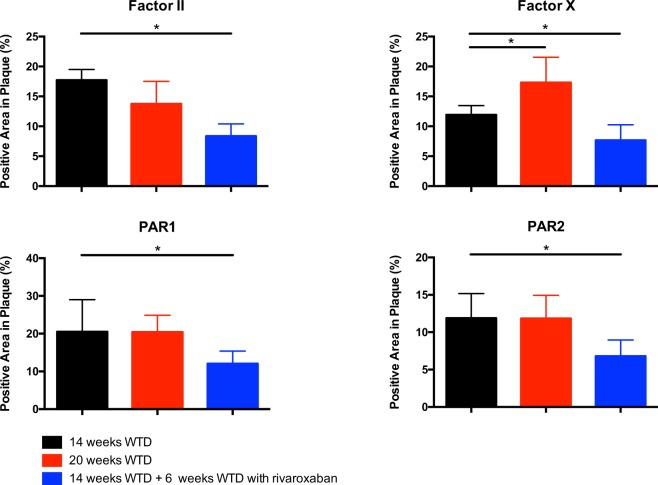
Diminished coagulation factor II and X and PAR1, PAR2 expression in atherosclerotic plaques of ApoE^−/−^ regression mice treated with rivaroxaban. (**A**) Quantitative analysis of FII after 14 weeks: 17.72% (12.05–19.52) and 20 weeks: 12.94% (10.58–16.55) vs 8.67% (7.11–10.15), (**B**) factor X after 14 weeks: 11.94% (8.11–13.48) and 20 weeks: 18.67% (14.15–20.11) vs 6.98% (5.65–10.35). (**C**) PAR-1 after 14 weeks: 20.52% (14.53–29.03) and 20 weeks: 21.60% (16.53–23.90) vs 11.34% (9.01–15.89). (**D**) PAR-2 after 14 weeks: 11.90% (8.52–15.18) and 20 weeks: 11.32% (9.54–13.45) vs 6.20% (5.54–7.45). All data were median (IQR), *p < 0.05 vs 14 weeks, n = 10 for each group.

IHC staining revealed a significant reduction of thrombin (−51%, *p* < *0.01*) and FX (−42%, *p* = *0.02*) in plaques of mice receiving rivaroxaban from 14 to 20 weeks, whereas levels of thrombin inside the plaque of WTD mice remained unchanged and FX levels significantly increased. In contrast, FVIIa remained unaffected in both groups (data not shown). Additionally, targeting FXa was associated with a significantly reduced expression of PAR1 (−45%, *p* < *0.01*) and PAR2 (−48%, *p* = *0.01*) in atherosclerotic lesions as compared to WTD from 14 to 20 weeks (+5%, *p* = *0.90* and −5%, *p* = *0.96*), respectively (Fig. 6).

## Discussion

In this study we show that treatment with the FXa inhibitor rivaroxaban not only decreases the onset and progression of atherosclerosis but also induces regression of already developed atherosclerotic lesions in ApoE^−/−^ mice. These findings were accompanied by increased plaque stability and decreased macrophages, the latter indicative of reduced inflammation.

Potential mechanisms that led to a reduced progression and regression of atherosclerosis could involve reduced PAR2 activation by FXa. Besides pre-clinical studies showing the anticoagulant properties of rivaroxaban, inhibition of FXa by rivaroxaban may reduce inflammation in a PAR-2 dependent matter. Zhou *et al*. used a low dose of rivaroxaban (27.3 ng/mL in plasma) in ApoE^−/−^ mice and observed no change in atherosclerotic volume. However, the expression of inflammatory cytokines, such as TNF-α, IL-6 and MCP-1, decreased upon rivaroxaban treatment and stabilized the plaque^16^. Another study that used a low dose of rivaroxaban (28.5 ng/mL in plasma) was able to attenuate progression of atherosclerosis by roughly 25%, as well as reduced inflammation, as reflected by decreased IL-1β, MCP-1, and TNF-α mRNA and protein levels^17^. In our mice study rivaroxaban plasma levels were on average 210 ng/mL, comparable to the human therapeutic levels and 10 times higher compared to the two previous studies. With the higher rivaroxaban plasma levels, the decrease in atherosclerosis progression was more pronounced, moreover inhibition of FXa induced regression of atherosclerosis in the carotid arteries after 6 weeks of anticoagulant treatment. The increased impact of rivaroxaban on both progression and regression of atherosclerosis can potentially be explained by the higher dose of rivaroxaban applied in our model. However, we cannot rule out other pathways involved. Thrombin is also reduced in our model due to FXa inhibition, and is known to play a role in atherogenesis in a PAR1 mediated manner^14,15,18–21^. Inhibition of thrombin with melagatran for 22 weeks in 30 weeks old ApoE^−/−^ mice reduced the progression of atherosclerosis, which was accompanied by significant thicker fibrotic cap thickness and smaller necrotic core, but had no effects on macrophages in the plaque^15,22^. However, a reduction of macrophages was observed upon inhibition of thrombin with dabigatran in other studies^14,19,21^. Although it is difficult to compare the effects of direct FXa inhibition with thrombin inhibition due to different concentrations of anticoagulants used, it is tempting to speculate about the differences on how they affect atherosclerosis. Targeting FXa induces regression of atherosclerosis and enhances plaque stability, whereas effects of thrombin inhibition might be more limited. These potential differences might be explained by the additional inhibition of PAR2 signaling upon targeting FXa along with PAR1, whereas direct inhibition of thrombin only decreases PAR1 activation. This is supported by the diminished atherosclerotic burden and increased plaque stability observed in ApoE^−/−^:PAR2^−/−^ mice^23^. This atheroprotective effect could be reversed by transfusion of bone marrow derived hematopoietic cells, most likely mainly PAR-2 containing macrophages. Hara *et al*. also showed that FXa as well as a PAR-2 activating peptide induced inflammatory mechanisms and increased lipid uptake in these macrophages. This mechanistic link between FXa inhibition and reduced macrophage trafficking and activity to and in the atherosclerotic lesion may provide an explanation for the reduced expression of PAR-1 and -2 in atherosclerotic lesions in our study.

The protective effects of FXa inhibition, and the subsequent decrease in FXa-PAR2 signaling, resulting in reduced macrophages in atherosclerotic plaques as described in our study, are in line with the phenotype of the ApoE^−/−^:PAR2^−/−^ mice. Given the presence of PAR-receptors on macrophages^24^, reduced macrophages in atherosclerotic plaques could be an important contributor to the observed decrease in PAR-receptors in atherosclerotic plaques.

In conclusion, this study suggests the involvement of FXa in atherogenesis and that direct inhibition of FXa stimulates regression of atherosclerotic plaques. The clinical relevance of these findings merits further studies.
